# Chronic ghrelin administration suppresses IKK/NF‐κB/BACE1 mediated Aβ production in primary neurons and improves cognitive function via upregulation of PP1 in STZ‐diabetic rats

**DOI:** 10.1002/alz70855_101492

**Published:** 2025-12-23

**Authors:** louyan Ma, Qiumin Qu

**Affiliations:** ^1^ Xi'an No.9 Hospital, Xi'an, ShaanXi, China; ^2^ The First Affiliated Hospital of Xi'an Jiaotong University, Xi'an, Shaanxi, China

## Abstract

**Background:**

To investigate the molecular mechanism of the brain‐gut peptide Ghrelin in improving cognitive function and attenuating Aβ deposition in hippocampal neurons.

**Method:**

A classical STZ‐induced diabetic rat model was established using 6‐8 weeks old, which were divided into normal control group, diabetes mellitus (DM) group, DM+Ghrelin group, DM+Ghrelin+ Dlys3‐GHRP‐6 (DG) (GHSR‐1a inhibitor) group. The MWM test was performed to test the learning and memory ability at the 12th week. The ultrastructural changes of hippocampal neurons were observed under electron microscopy. ELISA was used to detect the expression of MDA, ROS and Aβ in hippocampal neurons. Western blot was used to detect the expressions of GSHR‐1a, NF‐κB complex and BACE1. Co‐immunoprecipitation was used to detect the binding level of NF‐κB to BACE1 promoter. The hippocampal neurons of rats were cultured in primary to simulate the high glucose environment in vivo, and the cultures were intervened with high glucose, Ghrelin+high sugar, Ghrelin+DG+ high sugar, Ghrelin+high sugar+SC‐514, and Ghrelin+high glucose+BAY11‐7082, respectively. ELISA was used to detect the expressions of MDA, ROS and Aβ.

**Result:**

The DM group showed a decrease in MWM testing ability, the expression of MDA, ROS, and Aβ in the hippocampus increased, the expression of GHSR‐1a and PP1 decreased significantly, the IKK/NF‐κB/BACE1 pathway was activated, and neuronal apoptosis increased. The expression of PP1 in the hippocampus of the DM+ghrelin group was significantly increased, the IKK/NF‐κB/BACE1 pathway was inhibited, the deposition of Aβ was reduced, and the expressions of ROS and MDA were decreased. This effect is reversed by GHSR‐1a receptor antagonists. Similarly, high‐glucose‐treated primary cultured hippocampal neurons also exhibit low expression of GHSR‐1a and PP1. The expressions of *p*‐IKKβ, *p*‐p65, BACE1 and APP in hippocampal neurons decreased significantly after Ghrelin intervention, and increased significantly when GHSR‐1a and IKKβ phosphorus‐specific inhibitors were added. The expressions of *p*‐IKKβ, *p*‐p65, BACE1 and APP in hippocampal neurons decreased after the addition of IκBα phosphorylation inhibitors.

**Conclusion:**

Ghrelin binds to its receptor GHSR‐1a and inhibits the activation of NF‐κB, thereby inhibiting the expression of BACE1, reducing Aβ deposition, attenuating oxidative stress, and improving the learning and memory ability of diabetic rats.

